# Recent Advances in Nickel Catalysts with Industrial Exploitability for Copolymerization of Ethylene with Polar Monomers

**DOI:** 10.3390/polym16121676

**Published:** 2024-06-12

**Authors:** Ying Wang, Jingjing Lai, Rong Gao, Qingqiang Gou, Bingyi Li, Gang Zheng, Randi Zhang, Qiang Yue, Zhihui Song, Zifang Guo

**Affiliations:** Department of Polyethylene, SINOPEC (Beijing) Research Institute of Chemical Industry Co., Ltd., Beijing 100013, China; laijj.bjhy@sinopec.com (J.L.); gaor.bjhy@sinopec.com (R.G.); gouqq.bjhy@sinopec.com (Q.G.); liby.bjhy@sinopec.com (B.L.); zhenggang.bjhy@sinopec.com (G.Z.); zhangrd.bjhy@sinopec.com (R.Z.); yueq.bjhy@sinopec.com (Q.Y.); songzhh.bjhy@sinopec.com (Z.S.)

**Keywords:** polyethylene, polar monomer, functional polyethylene, nickel catalysts, coordination polymerization, high-temperature polymerization

## Abstract

The direct copolymerization of ethylene with polar monomers to produce functional polyolefins continues to be highly appealing due to its simple operation process and controllable product microstructure. Low-cost nickel catalysts have been extensively utilized in academia for the synthesis of polar polyethylenes. However, the development of high-temperature copolymerization catalysts suitable for industrial production conditions remains a significant challenge. Classified by the resultant copolymers, this review provides a comprehensive summary of the research progress in nickel complex catalyzed ethylene-polar monomer copolymerization at elevated temperatures in the past five years. The polymerization results of ethylene–methyl acrylate copolymers, ethylene-*tert*–butyl acrylate copolymers, ethylene–other fundamental polar monomer copolymers, and ethylene–special polar monomer copolymers are thoroughly summarized. The involved nickel catalysts include the phosphine-phenolate type, bisphosphine-monoxide type, phosphine-carbonyl type, phosphine-benzenamine type, and the phosphine-enolate type. The effective modulation of catalytic activity, molecular weight, molecular weight distribution, melting point, and polar monomer incorporation ratio by these catalysts is concluded and discussed. It reveals that the optimization of the catalyst system is mainly achieved through the methods of catalyst structure rational design, extra additive introduction, and single-site catalyst heterogenization. As a result, some outstanding catalysts are capable of producing polar polyethylenes that closely resemble commercial products. To achieve industrialization, it is essential to further emphasize the fundamental science of high-temperature copolymerization systems and the application performance of resultant polar polyethylenes.

## 1. Introduction

Polyethylene, currently available in hundreds of different grades commercially, is the most important and ubiquitous polymeric material [1,2,3,4]. The incorporation of polar functional groups into polyethylenes can improve their adhesion, dyeability, printability, barrier properties, rheology, degradability, compatibility, and other characteristics [5,6,7,8,9,10]. And even a small amount of polar monomer containing (<1%) is sufficient to impact the material performance [11,12]. However, the production of polar polyolefins has been an challenging issue in the field of olefin polymerization for a long time [13,14,15,16]. The manufacturing of polar polyethylenes in the industry is primarily achieved through multi-step post-functionalization and free-radical polymerization [17,18]. Post-functionalization strategy enables the production of copolymers that have tunable selectivity and good controllability, while the application of the product is restricted by limitations such as tedious reaction steps, low grafting efficiency, and the occurrence of side reactions [18]. Therefore, commercial free-radical copolymerization has been developed to overcome the abovementioned problems. Unfortunately, this method also has its specific drawbacks, including extremely harsh reaction conditions and excessive amounts of polar monomers in the resultant copolymer chains [17]. For instance, the ethylene–methyl acrylate (EMA) copolymers are typically produced under high temperatures (150–300 °C) and high pressures (150–300 MPa) [19]. In contrast, the gradually developing transition metal-catalyzed coordination insertion polymerization of ethylene and polar monomers delegates the most straightforward and promising approach for functionalizing polyethylenes.

For the coordination copolymerization, single-site catalysts have been frequently used to conduct the copolymerization of olefins and polar monomers. Finely controlled polymer microstructures can be achieved in this process. By optimizing the micro-environment of the active metal center, copolymers with a controllable chain topology and polar monomer insertion rate can be obtained under mild conditions. Early-transition metal-catalyzed olefin polar copolymerization has been constantly advanced [20,21]. Group IV transition metal (Ti, Zr, and Hf) catalysts are widely used for the copolymerization of ethylene and special polar monomers (with the polar group and C=C bond spaced with methylene) with high performance, whereas these oxophilic catalysts can readily form stable chelates with heteroatoms in polar monomers, resulting in reduced catalyst activity or even deactivation [22]. Consequently, they are quite helpless in the copolymerization of ethylene with fundamental polar monomers (with polar groups directly linked to the C=C bond). Certain protective strategies have been developed to mitigate the poisoning effect, for example, the prior complexation of polar monomers with a large amount of strong Lewis (methylaluminoxane or alkyl aluminum) [23,24], using a bulky group to shield the polar group and ensure that the active center of the catalyst is blocked [25], and employing polar monomers with long methylene chains between the polar group and the C=C bond [26]. It is therefore early metal catalytic systems for ethylene–polar monomer copolymerization which still suffer from complex operations and confined comonomer scopes.

Late-transition metal catalysts have gained impressive achievements in copolymerization for their good tolerance towards polar monomers. Owing to their weak electrophilicity and oxophilicity, Ni and Pd catalysts enable the direct copolymerization of ethylene with both fundamental polar monomers and special polar monomers [27,28,29]. Most related studies have focused on the use of Pd catalysts [30,31]. Compared to Ni catalysts bearing similar ligand structures, Pd catalysts typically demonstrate better tolerance towards polar groups. As a result, they are able to accommodate a wider range of polar monomer substrates, produce copolymers with higher molecular weight, and exhibit better control over the insertion rate of polar monomers. Pd catalysts are generally capable of catalyzing the copolymerization of ethylene with bulk polar monomers such as acrylate, acrylic acid, acrylonitrile, vinyl acetate, etc., while Ni catalysts are more limited to the special polar monomers [32,33,34]. Nevertheless, despite their good performance in copolymerization processes, the high cost and relatively low activity of Pd catalysts severely restrict their practical utility. In contrast, nickel catalysts are more appealing for industrial applications due to their greater economy, larger earth abundance, and higher catalytic activity in ethylene homopolymerization [35,36,37].

At the end of the 20th century, Grubbs et al. reported a series of neutral nickel catalysts that gave high molecular weight polymers and showed substantial tolerance towards polar agents [38,39]. These investigations have aroused widespread interest in developing effective nickel catalysts for the copolymerization of ethylene and polar monomers [27,35]. However, a significant limitation of its industrialization is the high-temperature catalytic performance of nickel catalysts. Industrial polyolefin production processes, such as slurry, gas phase, and solution polymerization, are generally carried out at temperatures above 70 °C to prevent reactor fouling and ensure continuous production [40]. Nevertheless, the majority of previous reports on the copolymerization of ethylene with polar monomers have been carried out at low temperatures (<80 °C) [7,28,41]. The deactivation mechanism of catalysts at high temperatures can be quite complex, with several processes potentially occurring simultaneously. The formation of metal-H species of salicylaldimine type catalysts may undergo reductive elimination to a zero-valent metal [42,43]. In the case of α-diimine type and bis(imino)pyridine type catalysts, the rotation of *N*-aryl moieties in the ligands may lead to catalyst decomposition [44]. Additionally, the σ-coordination of polar groups at the active metal center can cause a poisoning effect [2]. The copolymerization performance of nickel catalysts at elevated temperatures is currently receiving increasing attention from researchers [45,46,47]. Recently, some nickel catalysts have been reported to be involved in high-temperature ethylene copolymerization. Chen et al. have summarized the representative olefin–polar monomer coordination copolymerization catalytic systems with potential industrial applications based on polymerization processes [48]. In addition, to encourage industrial development prospects, it is also essential to summarize the copolymerization properties and resultant copolymer characteristics for each specific product. This will facilitate the direct comparisons between different nickel catalysts. Thus, it is expected that the screening or optimizing of desirable catalysts for the production of a particular product will be accelerated.

**Figure 1:**
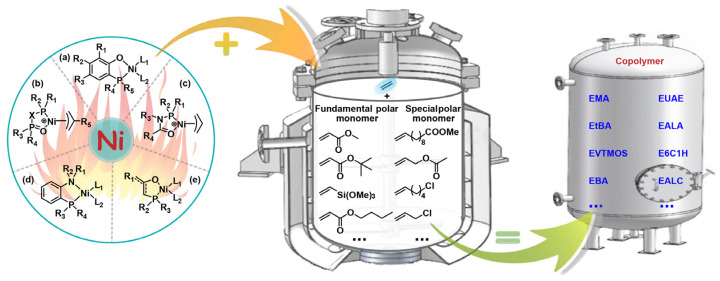
Typical structures of nickel catalysts for the synthesis of ethylene–polar monomer copolymerization: (**a**) phosphine-phenolate type; (**b**) bisphosphine-monoxide type; (**c**) phosphine-carbonyl type; (**d**) phosphine-benzenamine type; and (**e**) phosphine-enolate type.

This contribution reviews the research on the copolymerization of ethylene and polar monomers by using nickel catalysts at elevated temperatures in the past five years. Classified by the resultant copolymers, the relevant catalyst structures and copolymerization results are summarized. Note that there are two main types of comonomers involved in this review, i.e., fundamental polar monomers, and special polar monomers. Special polar monomers, characterized by the remote positioning of polar groups, are relatively easy to copolymerize with ethylene compared to the challenging fundamental polar monomers [22]. Based on a comb of the related literature, copolymer products are classified into four types: (1) EMA copolymers; (2) ethylene-*tert*–butyl acrylate (EtBA) copolymers; (3) ethylene–other fundamental polar monomer copolymers, such as ethylene–vinyltrimethoxysilane (EVTMOS) copolymers and ethylene-*n*–butyl acrylate (EBA) copolymers; (4) ethylene–special polar monomer copolymers, such as ethylene–methyl 10-undecenoate (EUAE) copolymers, ethylene–allyl acetate (EALA) copolymers, ethylene-6-chloro-1-hexene (E6C1H) copolymers, and ethylene–allyl chloride (EALC) copolymers. Bidentate nickel complexes of the phosphine-phenolate type, bisphosphine-monoxide type, phosphine-carbonyl type, phosphine-benzenamine type, and the phosphine-enolate type have been used to copolymerize ethylene with polar monomers (Figure 1). To provide a clear summary and comparison, the scopes of suitable polar monomer substrates and key copolymerization parameters obtained at or above 80 °C are listed. These parameters include catalytic activity, molecular weight, molecular weight distribution, melting point, and polar monomer incorporation ratio. The summarized data in this overview are expected to be convenient for the future design of desirable nickel catalysts for ethylene–polar monomer coordination copolymerization. In the final part of this contribution, the primary approaches for developing high performance nickel catalysts are outlined. Moreover, the challenges and promising directions of nickel catalysts with industrial exploitability for ethylene–polar monomer copolymerization are proposed.

## 2. Nickel Catalysts for Synthesizing EMA Copolymers

**Figure 2:**
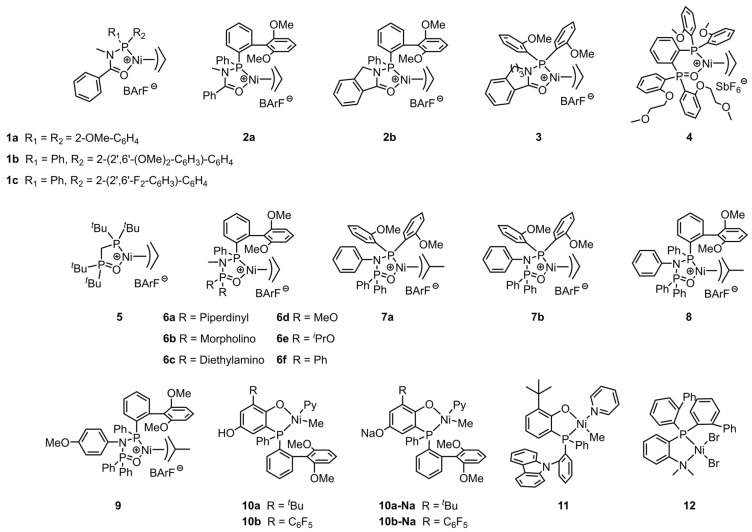
Chemical structures of phosphine-carbonyl Ni catalysts **1**–**3**, bisphosphine-monoxide Ni catalysts **4**–**9**, phosphine-phenolate Ni catalysts **10**–**11**, and phosphine-benzenamine Ni catalyst **12** for synthesizing EMA copolymers at elevated temperatures.

Commercial EMA copolymers are produced through high-temperature and high-pressure polymerization. Most of the brands currently available on the market are manufactured by companies such as DuPont (Wilmington, DE, USA), Westlake (Houston, TX, USA), and ExxonMobil (Irving, TX, USA). The MA content in the copolymers is about 10–30 wt%, with a melting point of approximately 80 °C. These products exhibit superior thermal stability, excellent flexibility and elasticity, good polymer compatibility, and easy processing properties. As a result, they are widely used in packaging, medical and health equipment, electronic and electrical equipment, automotive parts, and other related fields [49,50,51]. The coordinated copolymerization of ethylene with MA by nickel catalysts under industrial conditions has been explored. Typically, phosphine–carbonyl palladium and nickel catalysts exhibit ethylene oligomerization properties due to the weak coordination nature of C=O [52]. In 2020, Jian et al. proposed the N-bridged strategy to address the oligomerization issue [53]. The C-bridge between the C=O and phosphorus atom of the ligand was displaced by the N-bridge and a novel family of N-bridged phosphine–carbonyl Ni catalysts (**1a**–**c**) were synthesized (Figure 2). Although these catalysts demonstrated improved ethylene (co)polymerization properties, further development is needed for practical use in terms of copolymerization activity and copolymer molecular weight (Table 1, Entries 1–3). NMR spectroscopy results indicated that MA monomers are distributed within the chain and at the chain ends. Additionally, the influence of electronic and steric effects on the copolymerization performance of N-bridged phosphine–carbonyl Ni catalysts have been investigated by Tan et al. Compared to catalyst **2a**, catalyst **2b** bearing the cyclic backbone structure showed higher copolymerization activity (Act. = 40 kg mol^−1^ h^−1^) at the expense of the copolymer molecular weight and polar monomer incorporation ratio (Table 1, Entries 4 and 5) [54]. According to these findings, Jian’s team further employed the cyclizing strategy to develop high performance N-bridged phosphine-carbonyl Ni catalyst. Phosphine–carbonyl catalyst **3** bearing a seven-membered ring structure was synthesized for ethylene (co)polymerization [55]. The catalytic performance improved with the increase in ring size, and catalyst **3** achieved the MA incorporation ratio of 4.4% (Table 1, Entry 6). The high electron density around nitrogen atoms derived from methylene destabilizes the coordination of the carbonyl group to the central metal metal center. Consequently, the improved coordination strength and steric hindrance are responsible for the superior performance of **3**.

In addition to the upgrading of the phosphine–carbonyl Ni catalyst for ethylene and MA copolymerization, another classic [P^O] complex, namely, bisphosphine–monoxide Ni catalysts, has also received much attention. Previous research conducted by Do and co-workers has revealed that cation–tunable olefin polymerization catalysts could be realized by installing polyethylene glycol chains into the ligand framework [56,57]. Catalyst systems containing optimized secondary cation metal ions typically show improved polymerization activities and yield polyethylenes with high molecular weights. Based on these findings, the catalyst **4** bearing o-(2-methoxyethoxy)phenyl substituent on the P=O moiety was designed by their team to further explore the secondary ion effect in the copolymerization of ethylene and MA [58]. The copolymerization results indicated that the introduction of Li+ to catalyst **4** system led to obvious improvements in terms of catalytic activity and the polar monomer incorporation ratio compared to **4** alone (Table 1, Entries 7–9). The metal binding studies found that the promotion of catalytic performance is attributed to the formation of 1:1 nickel:lithium species. Compared to the **4**/Li^+^ catalytic system, the **4**/Na^+^ system showed approximative activity and molecular weight while displaying a dramatic increase in the MA incorporation ratio (up to 8.1%).

The bridging type between two phosphorus atoms on the ligand backbone has been found to significantly affect the catalytic performance of the complexes. In 2017, Chen et al. reported an imine-bridged bisphosphine-monoxide Ni catalyst, which is the first example of a nickel catalyst capable of catalyzing the copolymerization of ethylene with a variety of basic polar monomers [59]. This exceptional result is attributed to the small bite angle of P-Ni-O on the five-membered metallacycle and good ligand rigidity. Enlightened by this work, Nozaki et al. applied the methylene-bridged bisphosphine-monoxide bidentate ligand structure to nickel-based catalysts and successfully prepared the single crystal of **5** [60]. The single crystal structure revealed that the P-Ni-O bite angle in the five-membered metal ring is close to 90°. In the presence of NaBArF, the in situ generated (ligand/Ni(COD)_2_/PhCl) **5** can catalyze the copolymerization of ethylene/propylene with allyl acetate (ALA)/3-butenyl acetate/MA at 80 °C, while, interestingly, the single component of the cationic 3-allylnickel complex failed to conduct the copolymerization, and this issue could be solved by introducing a strong Lewis acid to the catalytic system [61]. The copolymerization results indicated that the copolymerization activity, copolymer molecular weight, and polar monomer incorporation ratio using catalyst **5** are all at a low level (Table 1, Entry 10). Subsequently, Chen and his co-workers prepared imine-bridged bisphosphine-monoxide catalysts **6a**–**f** with different substituents on the ligand for ethylene (co)polymerization (Table 1, Entries 11–16) [62]. In the absence of any cocatalyst, the copolymerization of ethylene with MA-generated polar polyethylenes with moderate molecular weights (*M*_n_ = 3.9–9.9 kg mol^−1^) and comonomer incorporation ratios (2.1–3.7%).

For bisphosphine-monoxide Ni catalysts, the catalytic properties are not only affected by the type of bridge on the ligand backbone, but also by the labile ligand [63]. Wang and his co-workers developed a series of novel 2-methylallyl-based nickel complexes (**7a**, **8** and **9**) to improve the chain initiation efficiency [64]. The μ-allyl-moiety was expected to modulate the electronic and steric effects, as well as the coordination environment around the active metal center, thereby modulating the copolymerization performance. Compared to its corresponding allyl-based analogue **7b**, the 2-methylallyl-based catalyst **7a** is more active (Act. = 2–17.5 kg mol^−1^ h^−1^) in the copolymerization of ethylene with MA at 80 °C (Table 1, Entries 17 and 18). Polar polyethylenes with moderate molecular weights (*M*_n_ = 4.2–10.1 kg/mol) and MA incorporation ratios (2.2–7.0%) were obtained during the copolymerization.

Slurry and gas phase processes using supported catalysts are the predominant methods for the production of polyolefins [65,66]. However, there are currently only a few studies on the application of heterogeneous catalysts for the synthesis of polar polyolefin [67,68,69]. In 2022, Chen et al. developed a universal ion anchoring strategy and prepared supported phosphine-phenolate Ni catalysts to copolymerize ethylene with diverse polar monomers [70]. Various solid supports, such as MgO, ZnO, Al_2_O_3_, TiO_2_, and SiO_2_, were employed to make this strategy more versatile. The MgO-supported nickel catalysts showed splendid copolymerization ability. The copolymerization of ethylene with MA by **10a-Na-MgO** produced polar polyethylenes with exceptionally high molecular weight (*M*_n_ = 101 kg mol^−1^) and copolymerization activity (Act. = 260 kg mol^−1^ h^−1^). These results are significantly better than those obtained from homogeneous catalyst **10a-Na** (Table 1, Entries 22 and 23). The outstanding performance of the supported catalysts derives from the enhanced interaction between the catalyst and the support, as well as the good tolerance towards polar monomers achieved by the simple and fast ion anchoring strategy. Additionally, another remarkable advantage of this method is that the resultant polymers are free-flowing particles that do not adhere to the surface of the reactor. This characteristic is highly beneficial for large-scale industrial continuous production processes.

Similar to the aforementioned bisphosphine-monoxide Ni catalyst **4**, installing polyethylene glycol chains into the phosphine-phenolate ligand has been proven to be an effective strategy to construct the cation-tunable ethylene (co)polymerization by forming secondary metal binding [71,72,73]. Moreover, another method for achieving cation-tunable polymerization was realized by assigning the coordinated N atom to the ligand. Zou et al. designed and synthesized a phosphine-phenolate Ni catalyst **11** with a nitrogen atom on the phosphine side of the ligand [74]. Catalyst **11** generated MA-functionalized polyethylenes with moderate catalytic activity, high molecular weight, and low MA incorporation ratio (Table 1, Entry 25). A phosphine–benzenamine Ni catalyst **12** was synthesized by Cai et al. for the direct copolymerization of ethylene or norbornene (NBE) with MA [75]. Polar polyethylenes with high molecular weight (*M*_n_ = 19.8 kg mol^−1^) and an unprecedented MA incorporation ratio (15.5%) were obtained in a moderately active (Act. = 22.7 kg mol^−1^ h^−1^) catalytic copolymerization system (Table 1, Entry 26). It was considered that the presence of bulky substituents on the ligand framework was beneficial in improving the catalytic activity and thermostability of the complex.

**Table 1:** Ethylene and MA copolymerization results using nickel catalysts at elevated temperatures.

Entry	Cat.	*T*_p_ (°C)	*P* (atm)	*t* (min)	Act. (kg mol^−1^ h^−1^)	*M*_n_ (kg mol^−1^)	*Ɖ*	*X* (mol%)	*T*_m_ (°C)	Ref.
1	**1a**	80	8	360	0.8	0.5	1.2	3.3	78.9	[53]
2	**1b**	80	8	360	8.3	6	2.3	1.8	116.3	[53]
3	**1c**	80	8	360	6.7	1	2.0	1.2	109.5	[53]
4	**2a**	80	7.9	120	7.5	24.4	2.0	1.6	124	[54]
5	**2b**	80	7.9	120	40	17.9	2.1	1.1	126	[54]
6	**3**	80	8	360	0.3	- ^a^	- ^a^	4.4	95.5	[55]
7	**4**	80	27.2	120	1.8–28.3	2.2–5.2	1.4–1.8	0.66–1.8	- ^a^	[58]
8	**4** + Li^+^	80	27.2	120	7.3–81	2.5–6.2	1.5–1.9	0.52–4.5	- ^a^	[58]
9	**4** + Na^+^	80	27.2	120	7.1	2.9	1.1	8.1	- ^a^	[58]
10	**5**	80	19.7	2820	0.51	1.8	2.4	0.56	124.3	[60]
11	**6a**	80	8	360	1.2	4.3	2.8	3.0	112.1	[62]
12	**6b**	80	8	360	1.4	3.9	2.7	3.7	111.7	[62]
13	**6c**	80	8	360	2.6	4.8	2.2	2.8	110.1	[62]
14	**6d**	80	8	360	10.1	6.6	2.2	2.2	117.8	[62]
15	**6e**	80	8	360	2.9	6.2	2.4	3.1	113.9	[62]
16	**6f**	80	8	360	5.8	9.9	2.2	2.1	123.0	[62]
17	**7a**	80	7.9–29.6	180	2–17.5	4.2–8.5	2.1–2.2	2.2–7.0	112.8–123.0	[64]
18	**7b**	80	7.9–29.6	180	1.2–14.5	3.4–7.3	2.3–2.8	1.8–6.3	112.6–122.5	[64]
19	**8**	80	7.9	180	7.7	10.1	2.8	2.5	123.9	[64]
20	**9**	80	7.9	180	13.8	6.0	2.3	2.7	120.9	[64]
21	**10a**	80	8	30	36	29	1.9	1.1	123.9	[70]
22	**10a**-**Na**	80	8	30	48	36	1.8	1.5	128.6	[70]
23	**10a**-**Na**-**MgO**	80–140	8	30	96–260	56–101	2.6	0.8–2.5	120.9–125.6	[70]
24	**10b**-**Na**-**MgO**	80	8–30	30	124–456	46–147	1.8–2.9	0.5–2.9	123.3–130.8	[70]
25	**11**	80	8	30	80	10.6–16.1	2.1–2.2	2.3–2.4	118.8–119.4	[74]
26	**12**	80	19.7	30	22.7	19.8	1.8	15.5	- ^a^	[75]

^a^ Not reported.

## 3. Nickel Catalysts for Synthesizing EtBA Copolymers

**Figure 3:**
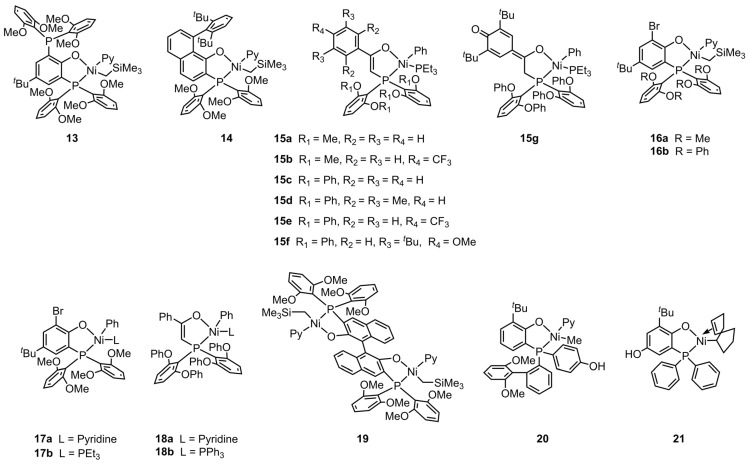
Chemical structures of phosphine-phenolate Ni catalysts **13**, **14**, **16**, **17,** and **19**–**21**, and phosphine-enolate Ni catalysts **15** and **18** for synthesizing EtBA copolymers at elevated temperatures.

The copolymerization of ethylene and *tert*–butyl acrylate (tBA) using nickel catalysts at elevated temperatures has been a focus of Agapie’s group in the past five years [76,77,78,79,80,81]. The authors synthesized thermally robust phosphine-phenolate Ni catalysts **13** and **14** for the copolymerization of ethylene with tBA (Figure 3) [76]. These catalysts exhibited high performance during the copolymerization, attributed to their ligands bearing either a bulk rotationally flexible phosphine group or a rigid aryl derivative. For example, with the catalysis of compound **13,** an unprecedented tBA incorporation ratio (12%) was achieved with high polymerization activity (Act. = 661 kg mol^−1^ h^−1^) during the copolymerization at 100 °C (Table 2, Entry 1). Crystal X-ray diffraction studies revealed that tBA insertion proceeds in a 2,1-insertion fashion. Moreover, the results of experimental kinetic analysis and density functional theory calculations indicated that the rate-limiting step of copolymerization is the monomer enchainment after tBA insertion. Thus, the copolymerization rates of ethylene with polar monomers are typically lower than those of ethylene homopolymerization. Furthermore, the research team also investigated the effect of secondary metal additives. Introducing the second metal of Al(O*^i^*Pr)_3_ in the catalyst **13** system in situ leads to an improvement in the activity (Act. = 1000 kg mol^−1^ h^−1^) for the copolymerization of ethylene and tBA (Table 2, Entry 3) [77]. The aluminum atom of Al(O*^i^*Pr)_3_ is likely weakly coordinated by ether groups in catalyst **13**. In addition, the phosphine-phenolate Ni catalyst **11** with Lewis acid modulation properties was also utilized for ethylene and tBA copolymerization with good performance (Table 2, Entry 4) [74].

Subsequently, Agapie et al. further designed a series of phosphine–enolate Ni catalysts **15a**–**g** with sandwich-like geometry to achieve higher copolymerization activity under elevated temperature conditions [78]. The efficient axial shielding effect of the four ether groups ortho to the phosphine is responsible for the conversion from an ethylene oligomerization SHOP-type catalyst to an excellent copolymerization catalyst [82]. The catalytic activity of **15c** is as high as 7700 kg mol^−1^ h^−1^ for the copolymerization of ethylene with tBA at 110 °C (Table 2, Entry 7), whereas the molecular weights (*M*_n_ = 1.2–6.6 kg mol^−1^) and the polar monomer incorporation ratios (0.3–2.8%) of the resultant copolymers by these catalysts still need further modulation. These in-chain ester functionalized copolymers show linear microstructures.

The β-H elimination caused by the polar monomers during the copolymerization is a crucial step that affects the microstructure and molecular weight of the resultant copolymer. However, relevant investigations are limited due to the difficulty in capturing relevant intermediates [42,83,84]. Agapie’s group disclosed a phosphine-phenolate Ni catalyst **16b**, which not only showed extraordinary copolymerization activity at elevated temperatures (Act. = 37,000 kg mol^−1^ h^−1^ at 130 °C), but also was suitable for investigating the polar monomer-induced β-H elimination process (Table 2, Entry 13) [79]. The single crystal of the Ni alkyl complex generated after tBA-induced β-H elimination and subsequent tBA insertion was obtained and analyzed. Hence, the alkyl chain release during the β-H elimination was confirmed, and a potential catalyst deactivation pathway was proposed. Note that both the acrylate-induced β-H elimination and catalyst decomposition can be suppressed by increasing ligand sterics. Moreover, to achieve an alternative routine for manufacturing the ethylene–acrylate copolymers, it is imperative to enhance the polar monomer incorporation ratio by rationally designing the ligands of these catalysts.

After elucidating the ligand substituent effect and copolymerization mechanism, the authors further investigated the influence of the labile ligand (L) on the copolymerization process [80]. Catalysts **17** and **18,** which have different labile ligands, were synthesized and compared. In situ studies disclosed that a weaker coordinating L resulted in faster chain propagation and more efficient catalyst initiation. Catalyst **18a,** bearing a pyridine ligand, exhibited four- to five-fold higher catalytic activity than **15c** with a PEt_3_ ligand (Table 2, Entries 7 and 16). The copolymerization activity value of ethylene and tBA by catalyst **18a** is as high as 24,000 kg mol^−1^ h^−1^, while the chain microstructure of the copolymer was found to be less affected by the labile ligands.

Inspired by the synergistic effect of multinuclear active sites in metalloenzymes, there has been a constant exploration of multi-metallic polyolefin catalysts [35,85,86]. For the olefin–polar monomer coordination copolymerization, the design of multi-metallic catalysts is envisioned to alleviate the low reactivity of the active center upon polar monomer insertion [15,87]. Very recently, Agapie et al. designed and synthesized the first binuclear nickel catalyst **19** for the copolymerization of ethylene and tBA [81]. The two active centers displayed distinct acrylate insertion rates. The first tBA insertion into one nickel center was faster than the subsequent insertion of tBA into the other. Additionally, NaBArF was introduced to modulate catalytic activity and copolymer properties (Table 2, Entries 18 and 19). A potential strategy for controlling chain enchainment during the polar polyethylene synthesis was thus proposed, i.e., cation exchange polymerization resulted from the dynamic exchange of Na^+^ at sub-stoichiometric ratios.

**Table 2:** Ethylene and tBA copolymerization results using nickel catalysts at elevated temperatures.

Entry	Cat.	*T*_p_ (°C)	*P* (atm)	*t* (min)	Act. (kg mol^−1^ h^−1^)	*M*_n_ (kg mol^−1^)	*Ɖ*	*X* (mol%)	*T*_m_ (°C)	Ref.
1	**13**	90–100	27.2	60–75	82–661	8.7–27	2.2–2.4	2.15–11.95	68.4–110.9	[76]
2	**14**	90–100	27.2	60	205–637	3.9–7.2	2–2.3	0.7–2.0	110.6–121.1	[76]
3	**13** + Al(O*^i^*Pr)_3_	90	27.2	25–58	510–1000	16.6–22.4	2.3–2.4	2.2–4.6	99–111	[76]
4	**11**	80–150	8	30	40–160	6.1–32.8	1.7–3.0	1.0–3.9	115.9–125.8	[74]
5	**15a**	90	27.2	30–60	190–490	1.2–1.8	1.9–2.1	1.6–2.8	77–113	[78]
6	**15b**	90	27.2	26	770	1.9	3.2	1.4	114	[78]
7	**15c**	90–110	27.2	6–56.6	1160–7700	2.7–4.4	2.4–2.8	0.3–1.4	118–125	[78]
8	**15d**	90	27.2	25	1090	3.3	2.1	0.7	116	[78]
9	**15e**	90	27.2	13–15	1340	4.6	2.2	0.5	122	[78]
10	**15f**	90	27.2	16–20	1850	6.6	2.3	0.6	123	[78]
11	**15g**	90	27.2	31–57	450–590	4.5–6.3	2.4–2.5	0.6–0.7	122–123	[78]
12	**16a**	90–110	27.2	17–60	440–1550	7.4–30.5	2.2–2.4	1.5–4.8	99–115	[83]
13	**16b**	90–130	27.2	2–31	5700–37,000	6–16.7	2.2–2.6	0.3–1.0	120–127	[83]
14	**17a**	90	27.2	24–60	440–1100	25–34	2.2–2.3	1.6–3.2	105–115	[84]
15	**17b**	90	27.2	60	120–210	15.9–27.4	2.3–2.6	1.7–3.5	104–114	[84]
16	**18a**	90–110	27.2–29.3	2–10	2900–24,000	2.7–4.8	2.2–2.6	0.3–1.6	112–124	[84]
17	**18b**	90	27.2	5	5600	5.7	2.1	0.5	122	[84]
18	**19**	90–110	27.2	60	93–240	14.3–29.3	2.8–3.7	1.9–6.1	96–115	[87]
19	**19** + NaBArF	90–110	27.2	26–60	410–920	5.9–14.1	2.4–3.8	0.7–1.1	109–121	[87]
20	**10a-Na-MgO**	80–140	8–30	30	100–1660	32–834	1.8–3.0	1–7.4	120.9–133.4	[70]
21	**10b-Na-MgO**	80	30	30	1260–4100	117–343	2.4–4.0	0.3–1.2	128.2–132.4	[70]
22	**10a/21-MgO**	80	8–30	30	160–350	0.9–13.3	11.5–28.5	0.2–1.4	125.0–132.1	[88]
23	**20/21-MgO**	80	8	30	400	1.58	34.4	0.7	129.9	[88]
24	**20-MgO/21-MgO**	80	8	30	600	1.64	34.2	0.6	118.7/133.9	[88]

The supported phosphine-phenolate Ni catalysts **10a-Na-MgO** and **10b-Na-MgO** developed using the ion anchoring strategy were used for the copolymerization of ethylene and tBA [70]. The process produced polar polyethylene that has high catalytic activity (Act. = 4100 kg mol^−1^ h^−1^), molecular weight (*M*_n_ = 834 kg mol^−1^), and tBA incorporation ratio (7.4%) (Table 2, Entries 20 and 21). Based on these excellent polymerization results, Chen and his co-workers further targeted the synthesis of polar bimodal polyolefins using the co-anchoring strategy [88]. Three synthetic routes were designed for the synthesis of polar bimodal polyolefins, including mixtures of homogeneous catalysts, separately supported heterogeneous catalysts, and a co-anchoring strategy supporting different homogeneous catalysts (**10a**, **20**, and **21**) on one solid carrier. The tensile properties of the polar bimodal polyethylenes obtained from the three catalytic systems were compared. It was found that the tensile properties of the polar bimodal polyethylenes prepared using the first two common methods were significantly weaker than the average tensile properties of the two fractions in the bimodal polyethylenes. This was attributed to poor miscibility between the two polar polyethylene fractions (the more branched/more polar low molecular weight fraction and the less branched/less polar high molecular weight fraction) which led to severe phase separation. In contrast, polar bimodal polyethylenes prepared using the co-anchoring strategy maintained the excellent tensile properties of the corresponding high molecular weight fraction. This happened because the co-anchoring strategy led to molecular-level entanglement and even co-crystallization of the two distinct fractions, thus decreasing the phase separation between them. These bimodal EtBA copolymers (tBA incorporation ratios of 0.2–1.4 mol %) were produced with high copolymerization activities (160–400 kg mol^−1^ h^−1^) at 80 °C. Additionally, the copolymers displayed excellent surface properties, dyeing properties, tensile properties, gas barrier properties, extrudability, and 3D printability (Table 2, Entries 22 and 23).

## 4. Nickel Catalysts for Synthesizing Copolymers of Ethylene and Other Fundamental Polar Monomers

Vinyltrimethoxysilane (VTMOS) is easier to copolymerize with ethylene than other fundamental polar monomers because it has lower toxicity for the catalyst. The functional polyethylenes containing silicon–oxygen bonds can serve as versatile processing additives [89,90]. Homogeneous imine-bridged bisphosphine-monoxide Ni catalysts **6b** and **6d,** as well as the heterogeneous phosphine-phenolate Ni catalyst **10a-Na-MgO,** have been reported for synthesizing ethylene and VTMOS copolymers at 80 °C (Table 3, Entries 1–3) [62,70]. The copolymerization results revealed that the supported catalyst **10a-Na-MgO** exhibited the highest catalytic activity (Act. = 104 kg mol^−1^ h^−1^), while catalyst **6b** gave polar polyethylenes with the largest VTMOS incorporation ratio (4.2%).

The production of commercialized EBA copolymers involves using free-radical polymerization at high temperatures and high pressures. These copolymers exhibited excellent flexibility, temperature resistance, and impact resistance. As a result, EBA copolymers are widely used in various industries such as wire and cable, adhesive, packaging film, toys, medical soft tubes, and extrusion coating [91,92]. The supported phosphine-phenolate Ni catalyst **10a-Na-MgO** was used for the synthesis of ethylene and *n*-butyl acrylate (BA)/2-methoxyethyl acrylate copolymers [70]. Copolymers with high molecular weights (*M*_n_ = 33–62 kg mol^−1^) were obtained with moderate polymeric activities (Act. = 60–76 kg mol^−1^ h^−1^), while the polar monomer incorporation ratios (1.2–1.5%) were found to be unsatisfactory (Table 3, Entries 4 and 5). The copolymerization of ethylene with *n*-butyl vinyl ether (BVE) at elevated temperatures was carried out using the N-bridged phosphine–carbonyl Ni catalysts **1b** and **1c** [53]. Unfortunately, the polymeric activities, molecular weights of copolymers, and BVE incorporation ratios are all at a low level and in need of being greatly improved (Table 3, Entries 6 and 7). The NMR spectroscopy of the copolymers indicated that BVE monomers were only located at the ends of polymer chains.

## 5. Nickel Catalysts for Synthesizing Copolymers of Ethylene and Special Polar Monomers

The decoration of polyolefins with long chain structure methyl 10-undecenoate (UAE) can significantly improve the surface properties and tensile properties of the material [93]. The single-site bisphosphine-monoxide Ni catalyst **6d**, phosphine-phenolate Ni catalyst **11**, as well as the supported heterogeneous phosphine-phenolate Ni catalyst **10a-Na-MgO,** have been employed in the synthesis of UAE functionalized polyethylenes (Table 4, Entries 1–3) [62,70,74]. Notably, **10a-Na-MgO** stands out for its high polymeric activities (up to 1440 kg mol^−1^ h^−1^) and the ability to generate copolymers with high molecular weights (*M*_n_ = 47–307 kg mol^−1^). Moreover, the bimodal ethylene and UAE copolymers were produced using the heterogeneous catalyst **20/21-MgO** developed by the co-anchoring strategy (Table 4, Entry 4) [88]. These bimodal copolymers possess excellent tensile properties due to the reduced phase separation between the two fractions. In addition, the copolymerization of ethylene and NBE derivatives has also been achieved using catalyst **6d** (Table 4, Entry 6). In addition, although the copolymerization of ethylene and ALA at 80 °C was achieved by using the N-bridged phosphine–carbonyl Ni catalysts **2a** and **2b** as well as the methylene-bridged bisphosphine-monoxide Ni catalyst **5**, the catalytic activities and the ALA incorporation ratios were somewhat unacceptable (Table 4, Entries 7–9) [54,60].

The introduction of chlorine atoms and hydroxyl groups into polyethylene chains endows the polymers with valuable potential for further reactions to obtain high-value-added materials [94,95,96,97]. The N-bridged phosphine–carbonyl Ni catalyst **2b,** bisphosphine-monoxide Ni catalysts **6b** and **6d,** and the heterogeneous phosphine-phenolate Ni catalyst **10a-Na-MgO** have been developed for the copolymerization of ethylene with chloro-substituted α-olefins under elevated temperatures (Table 4, Entries 10–14) [54,62,70]. The polymerization performance (Act. = 1900 kg mol^−1^ h^−1^, *M*_n_ = 268 kg mol^−1^) of the supported catalyst **10a-Na-MgO** in the copolymerization of ethylene with 6-chloro-1-hexene are superior by an order of magnitude to those in ethylene and allyl chloride copolymerization. Consequently, the distance between the polar group and the C=C bond almost has a decisive effect on the copolymerization activity and the molecular weight of the copolymer. The supported nickel catalyst **10a-Na-MgO** and phosphine-phenolate Ni catalyst **11** have been used to synthesize ethylene and 10-undecenol copolymers (Table 4, Entries 15 and 16) [74,88]. The supported **10a-Na-MgO** once again exhibited excellent copolymerization performance. In addition, polyethylenes decorated with 3-butenenitrile were also obtained at 80 °C by using the catalyst **10a-Na-MgO**, while the reaction activity and polymer molecular weight were found to be relatively low (Table 4, Entry 17).

**Figure 4:**
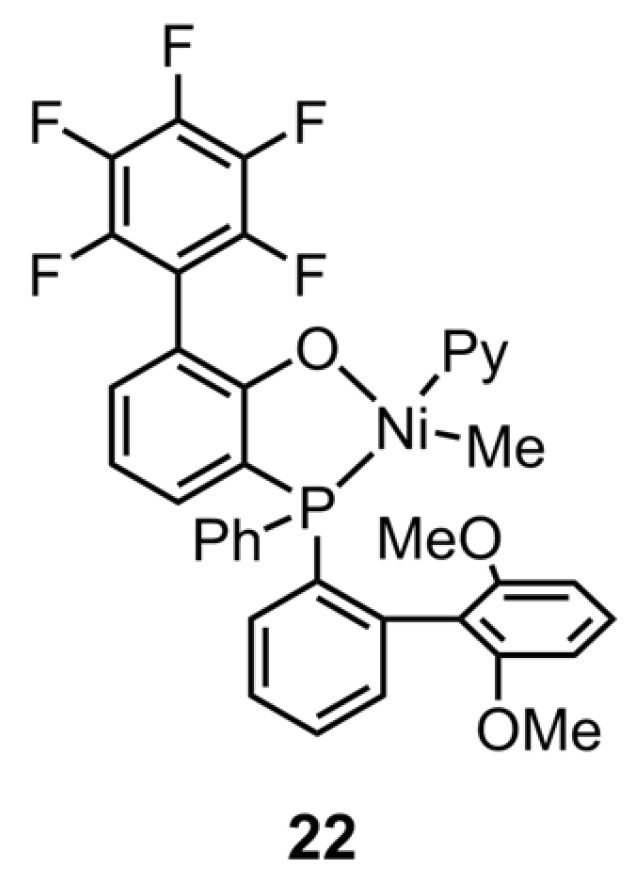
Chemical structure of phosphine-phenolate Ni catalyst **22** for synthesizing copolymers of ethylene and the special polar monomer at elevated temperatures.

In response to the issue of white pollution, there has been a long-standing search for polyethylenes with degradability [98,99,100]. A recent trend in research has focused on photodegradable linear HDPE-like materials that have keto groups decorated on the polyethylene backbone. Despite the fact that the non-alternating copolymers of ethylene and CO have been realized by the advanced neutral phosphine-phenolate Ni catalysts, their highly crystalline nature remains a barrier to their use as soft materials. Mecking’s team solved this problem by introducing NBE as the non-crystallizable comonomer to synthesize crystallinity-reduced in-chain keto-functionalized polyethylenes [101]. With the catalysis of **22** (Figure 4), the introduction of NBE allows for the tuning of the crystallinity of polar polyethylene with a minor decrease in its molecular weight (Table 4, Entries 18 and 19). Furthermore, these copolymers were characterized as soft materials with excellent ductility, making them suitable for use as film products.

**Table 4:** Ethylene and special polar monomer copolymerization results using nickel catalysts at elevated temperatures.

Entry	Cat.	Polar Monomer	*T*_p_ (°C)	*P* (atm)	*t* (min)	Act. (kg mol^−1^ h^−1^)	*M*_n_ (kg mol^−1^)	*Ɖ*	*X* (mol%)	*T*_m_ (°C)	Ref.
1	**6d**	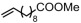	80	8	360	180	5.2	2.2	2.9	112.2	[62]
2	**10a-Na-MgO**	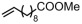	80–140	8	30	1800–2320	47–307	2.2–2.9	0.6–2.4	126.7–130.8	[70]
3	**11**	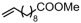	80–150	8	30	680–1440	5.5–30.8	2.3–2.6	0.8–2.0	117.5–124.3	[74]
4	**20/21-MgO**	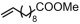	80–120	8–30	30	660–3920	4–8.8	3.7–13.3	0.5–1.3	126.4–132.2	[88]
5	**20-MgO/21-MgO**	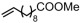	80	8	30	1200	2.11	34.4	0.5	125.6/131.1	[88]
6	**6d**	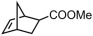	80	8	360	52	8.7	1.8	1.0	123.7	[62]
7	**2a**	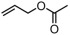	80	7.9	120	1.5	13.7	1.8	1.1	125	[54]
8	**2b**	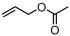	80	7.9	120	2.0	8.7	2.6	0.7	127	[54]
9	**5**	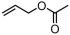	80	19.7–29.6	1440–2820	0.02–2.4	0.9–35	2.6–5.0	0.05–0.81	118.3–129.1	[60]
10	**6b**	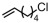	80	8	360	40	5.8	2.2	1.2	109.4	[62]
11	**6d**	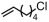	80	8	360	160	7.2	2.0	1.4	119.5	[62]
12	**10a-Na-MgO**	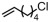	80	8	30	1900	268	2.1	0.7	129	[70]
13	**2b**	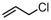	80	7.9	120	6.5	12.9	2.4	0.6	133	[54]
14	**10a-Na-MgO**	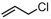	80	8	30	24	38	1.8	0.3	128.2	[70]
15	**10a-Na-MgO**	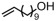	80	8	30	2060	292	2.3	0.7	129	[70]
16	**11**	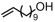	80–150	8	30	120–520	12.8–47.6	2.3–2.4	0.6–1.2	121.1–123.9	[74]
17	**10a-Na-MgO**	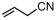	80	8	30	40	48	2.1	0.7	128.4	[70]
18	**22**	CO/NBE	100	10	75	53.6–109.6	29–43	1.7–2.0	0.7–1.1	97–126	[101]
19	**22**	CO	100	10	75	173	65	1.6	1.3	136	[101]

## 6. Conclusions and Outlook

Over the past five years, nickel catalysts have achieved encouraging progress in the field of ethylene and polar monomer copolymerization at elevated temperatures. Particularly, the incorporation ratios of MA and tBA have reached as high as 15.5% and 12%, respectively. These values closely resemble commercial products, which highlights that nickel catalysts are on the brink of industrialization. This contribution provides a comprehensive overview of the nickel catalysts employed for the high-temperature copolymerization of ethylene and polar monomers. The characteristics of copolymerization, including catalytic activity, molecular weight, molecular weight distribution, melting point, and polar monomer incorporation ratio, are collected to provide an intuitive assessment of each catalyst. The current research directions of nickel catalysts for high-temperature copolymerization primarily focus on three aspects. The first aspect involves the rational design of the catalyst structure, with effective strategies being adopted such as modifying the bridging atom at the ligand backbone, modulating the electronic and steric effects of the substituents, and utilizing the multinuclear synergistic effect, etc.. The second aspect refers to the introduction of extra additives to construct the secondary cation metal ion effect. Additionally, the heterogenization of single-site catalysts is considered another useful method to enhance the catalytic performance. This approach is exemplified by the ion anchoring strategy, which exhibits great potential in improving copolymerization activity.

Nickel catalysts for the copolymerization of ethylene and polar monomers at elevated temperatures have been constantly studied for their attractive industrialization prospects. However, despite the many remarkable copolymerization results that have been achieved using nickel catalysts, the bottleneck for future development still exists. Particularly, meeting the targets of catalytic activity, polar polyethylene molecular weight and polar monomer incorporation ratio simultaneously represent a significant obstacle towards further advancement. Moreover, in addition to accelerating the development of traditional polyolefins (ethylene–acrylate copolymers), further efforts are needed to excavate high-value-added polar polyethylenes with groundbreaking application scenarios. The challenges mentioned above demonstrate a higher demand for both basic scientific research and a practical exploration of nickel catalysts. The mechanism of ethylene–polar monomer copolymerization and catalyst deactivation at elevated temperatures needs to be clarified comprehensively. Furthermore, emphasizing the assessment of polymer application performance will accelerate the industrialization progress of functional polar polyethylene products.

## Figures and Tables

**Table 3:** Ethylene and other fundamental polar monomer copolymerization results using nickel catalysts at elevated temperatures.

Entry	Cat.	Polar Monomer	*T*_p_ (°C)	*P* (atm)	*t* (min)	Act. (kg mol^−1^ h^−1^)	*M*_n_ (kg mol^−1^)	*Ɖ*	*X* (mol%)	*T*_m_ (°C)	Ref.
1	**6b**	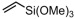	80	8	360	52	4.5	2.7	4.2	103.7	[62]
2	**6d**	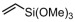	80	8	360	72	7.0	1.9	2.4	118.3	[62]
3	**10a-Na-MgO**	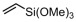	80	8	30	104	97	2.3	0.1	130.3	[70]
4	**10a-Na-MgO**	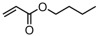	80	8	30	76	62	2.3	1.5	126.5	[70]
5	**10a-Na-MgO**	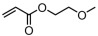	80	8	30	60	33	1.7	1.2	125.9	[70]
6	**1b**	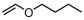	80	8	360	0.7	1.9	2.4	2.0	114.2	[53]
7	**1c**	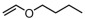	80	8	360	0.4	2.5	2.4	2.6	118.3	[53]

## References

[1] Hutley T.J., Ouederni M., Al-Ali AlMa’adeed M., Krupa I. (2016). Polyolefins-the history and economic impact. Polyolefin Compounds and Materials: Fundamentals and Industrial Applications.

[2] Chen C. (2018). Designing catalysts for olefin polymerization and copolymerization: Beyond electronic and steric tuning. Nat. Rev. Chem..

[3] Hustad P.D. (2009). Frontiers in olefin polymerization: Reinventing the world’s most common synthetic polymers. Science.

[4] Sauter D.W., Taoufik M., Boisson C. (2017). Polyolefins, a success story. Polymers.

[5] Zou C., Chen C. (2020). Polar-functionalized, crosslinkable, self-healing, and photoresponsive polyolefins. Angew. Chem. Int. Ed..

[6] Baur M., Lin F., Morgen T.O., Odenwald L., Mecking S. (2021). Polyethylene materials with in-chain ketones from nonalternating catalytic copolymerization. Science.

[7] Tan C., Zou C., Chen C. (2022). Material properties of functional polyethylenes from transition-metal-catalyzed ethylene-polar monomer copolymerization. Macromolecules.

[8] Tan C., Zou C., Chen C. (2022). An ionic cluster strategy for performance improvements and product morphology control in metal-catalyzed olefin-polar monomer copolymerization. J. Am. Chem. Soc..

[9] Mu H., Zhou G., Hu X., Jian Z. (2021). Recent advances in nickel mediated copolymerization of olefin with polar monomers. Coord. Chem. Rev..

[10] Tan C., Chen C. (2019). Emerging palladium and nickel catalysts for copolymerization of olefins with polar monomers. Angew. Chem. Int. Ed..

[11] Gong Y., Li S., Gong Q., Zhang S., Liu B., Dai S. (2019). Systematic investigations of ligand steric effects on alpha-diimine nickel catalyzed olefin polymerization and copolymerization. Organometallics.

[12] Chen Z., Leatherman M.D., Daugulis O., Brookhart M. (2017). Nickel-catalyzed copolymerization of ethylene and vinyltrialkoxysilanes: Catalytic production of cross-linkable polyethylene and elucidation of the chain-growth mechanism. J. Am. Chem. Soc..

[13] Na Y., Chen C. (2020). Catechol-functionalized polyolefins. Angew. Chem. Int. Ed..

[14] Gao J., Cai W., Hu Y., Chen C. (2019). Improving the flame retardancy of polyethylenes through the palladium-catalyzed incorporation of polar comonomers. Polym. Chem..

[15] Takano S., Takeuchi D., Osakada K., Akamatsu N., Shishido A. (2014). Dipalladium catalyst for olefin polymerization: Introduction of acrylate units into the main chain of branched polyethylene. Angew. Chem. Int. Ed..

[16] Ruenzi T., Mecking S. (2014). Saturated polar- substituted polyethylene elastomers from insertion polymerization. Adv. Funct. Mater..

[17] Mazzolini J., Boyron O., Monteil V., Gigmes D., Bertin D., D’Agosto F., Boisson C. (2011). Polyethylene end functionalization using radical-mediated thiol-ene chemistry: Use of polyethylenes containing alkene end functionality. Macromolecules.

[18] Boaen N.K., Hillmyer M.A. (2005). Post-polymerization functionalization of polyolefins. Chem. Soc. Rev..

[19] Du W., Zheng H., Li Y., Cheung C., Li D., Gao H., Deng H., Gao H. (2022). Neutral tridentate α-sulfonato-β-diimine nickel catalyst for (co)polymerizations of ethylene and acrylates. Macromolecules.

[20] Chen J., Gao Y., Marks T. (2020). Early transition metal catalysis for olefin-polar monomer copolymerization. Angew. Chem. Int. Ed..

[21] Schöbel A., Winkenstette M., Anselment T., Rieger B., Matyjaszewski K., Möller M. (2012). Copolymerization of alkenes and polar monomers by early and late transition metal catalysts. Polymer Science: A Comprehensive Reference.

[22] Nakamura A., Ito S., Nozaki K. (2009). Coordination-insertion copolymerization of fundamental polar monomers. Chem. Rev..

[23] Imuta J., Kashiwa N., Toda Y. (2002). Catalytic regioselective introduction of allyl alcohol into the nonpolar polyolefins: Development of one-pot synthesis of hydroxyl-capped polyolefins mediated by a new metallocene IF catalyst. J. Am. Chem. Soc..

[24] Terao H., Ishii S., Mitani M., Tanaka H., Fujita T. (2008). Ethylene/polar monomer copolymerization behavior of bis(phenoxy-imine)Ti complexes: Formation of polar monomer copolymers. J. Am. Chem. Soc..

[25] Toda T., Nakata N., Matsuo T., Ishii A. (2011). Synthesis and structures of dialkyl zirconium complexes with an OSSO-type bis(phenolate) ligand bearing a *trans*-1,2-cyclooctanediylbis(thio) unit. J. Organomet. Chem..

[26] Wang Y., Jiang L., Ren X., Guo F., Hou Z. (2021). Synthesis of bromine-functionalized polyolefins by scandium-catalyzed copolymerization of 10-bromo-1-decene with ethylene, propylene, and dienes. J. Polym. Sci..

[27] Mu H., Pan L., Song D., Li Y. (2015). Neutral nickel catalysts for olefin homo- and copolymerization: Relationships between catalyst structures and catalytic properties. Chem. Rev..

[28] Chen Z., Brookhart M. (2018). Exploring ethylene/polar vinyl monomer copolymerizations using Ni and Pd alpha-diimine catalysts. Accounts. Chem. Res..

[29] Zhang W., Waddell P.M., Tiedemann M.A., Padilla C.E., Mei J., Chen L., Carrow B.P. (2018). Electron-rich metal cations enable synthesis of high molecular weight, linear functional polyethylenes. J. Am. Chem. Soc..

[30] Zheng H., Qiu Z., Li D., Pei L., Gao H. (2023). Advance on nickel- and palladium-catalyzed insertion copolymerization of ethylene and acrylate monomers. J. Polym. Sci..

[31] Khan W.U., Mazhar H., Shehzad F., Al-Harthi M.A. (2023). Recent advances in transition metal-based catalysts for ethylene copolymerization with polar comonomer. Chem. Rec..

[32] Rünzi T., Fröhlich D., Mecking S. (2010). Direct synthesis of ethylene−acrylic acid copolymers by insertion polymerization. J. Am. Chem. Soc..

[33] Daigle J.-C., Piche L., Claverie J.P. (2011). Preparation of functional polyethylenes by catalytic copolymerization. Macromolecules.

[34] Friedberger T., Wucher P., Mecking S. (2012). Mechanistic insights into polar monomer insertion polymerization from acrylamides. J. Am. Chem. Soc..

[35] Ji G., Chen Z., Wang X., Ning X., Xu C., Zhang X., Tao W., Li J., Gao Y., Shen Q. (2021). Direct copolymerization of ethylene with protic comonomers enabled by multinuclear Ni catalysts. Nat. Commun..

[36] Li Q., Mu H., Jian Z. (2023). Facile access to diverse polyethylenes via neutral salicylaldiminato nickel catalysts. Polym. Chem..

[37] Zheng H., Li Y., Du W., Cheung C., Li D., Gao H., Deng H., Gao H. (2022). Unprecedented square-planar α-diimine dibromonickel complexes and their ethylene polymerizations modulated by Ni-phenyl interactions. Macromolecules.

[38] Wang C., Friedrich S., Younkin T.R., Li R.T., Grubbs R.H., Bansleben D.A., Day M.W. (1998). Neutral nickel(II)-based catalysts for ethylene polymerization. Organometallics.

[39] Younkin T.R., Conner E.F., Henderson J.I., Friedrich S.K., Grubbs R.H., Bansleben D.A. (2000). Neutral, single-component nickel (II) polyolefin catalysts that tolerate heteroatoms. Science.

[40] Ali E.M., Abasaeed A.E., Al-Zahrani S.M. (1998). Optimization and control of industrial gas-phase ethylene polymerization reactors. Ind. Eng. Chem. Res..

[41] Zhou G., Cui L., Mu H., Jian Z. (2021). Custom-made polar monomers utilized in nickel and palladium promoted olefin copolymerization. Polym. Chem..

[42] Song Z., Wang S., Gao R., Wang Y., Gou Q., Zheng G., Feng H., Fan G., Lai J. (2023). Recent advancements in mechanistic studies of palladium- and nickel-catalyzed ethylene copolymerization with polar monomers. Polymers.

[43] Nozaki K., Kusumoto S., Noda S., Kochi T., Chung L.W., Morokuma K. (2010). Why did incorporation of acrylonitrile to a linear polyethylene become possible? Comparison of phosphine-sulfonate ligand with diphosphine and imine-phenolate ligands in the pd-catalyzed ethylene/acrylonitrile copolymerization. J. Am. Chem. Soc..

[44] Rhinehart J.L., Brown L.A., Long B.K. (2013). A robust Ni(II) alpha-diimine catalyst for high temperature ethylene polymerization. J. Am. Chem. Soc..

[45] Wang Y., Gao R., Gou Q., Lai J., Zhang R., Li X., Guo Z. (2022). Developments in late transition metal catalysts with high thermal stability for ethylene polymerization: A crucial aspect from laboratory to industrialization. Eur. Polym. J..

[46] Mitchell N.E., Long B.K. (2019). Recent advances in thermally robust, late transition metal-catalyzed olefin polymerization. Polym. Int..

[47] Ma Z., Yang W., Sun W.-H. (2017). Recent progress on transition metal (Fe, Co, Ni, Ti and V) complex catalysts in olefin polymerization with high thermal stability. Chin. J. Chem..

[48] Tan C., Chen M., Zou C., Chen C. (2024). Potentially practical catalytic systems for olefin-polar monomer coordination copolymerization. CCS Chem..

[49] Gao Z., Wu Y., Xu L., Hao H., Wu Q., Xie H. (2018). Preparation and luminescent properties of Eu(III) organic complex and novel transparent ethylene-methyl acrylate copolymer (EMA) films doped with complexes. Opt. Mater..

[50] Ding J., Yue Z., Sun J., Zhou J., Gao J. (2016). Effect of ABS/PMMA/EMA ternary blending sequence on mechanical properties and surface glossiness. J. Polym. Eng..

[51] Cavodeau F., Viretto A., Otazaghine B., Lopez-Cuesta J.-M., Delaite C. (2017). Influence of colemanite on the fire retardancy of ethylene-vinyl acetate and ethylene-methyl acrylate copolymers. Polym. Degrad. Stabil..

[52] Behzadi S., Chi M., Pang W., Liang T., Tan C. (2020). Camphor-based phosphine-carbonyl ligands for Ni catalyzed ethylene oligomerization. New J. Chem..

[53] Cui L., Jian Z. (2020). A N-bridged strategy enables hemilabile phosphine-carbonyl palladium and nickel catalysts to mediate ethylene polymerization and copolymerization with polar vinyl monomers. Polym. Chem..

[54] Zhu N., Liang T., Huang Y., Pang W., Chen M., Tan C. (2021). Influences of ligand backbone substituents on phosphinecarbonylpalladium and -nickel catalysts for ethylene polymerization and copolymerization with polar monomers. Inorg. Chem..

[55] Cui L., Chu Y.-K., Liu D.-J., Han Y.-F., Mu H.-L., Jian Z.-B. (2022). Enhancement on hemilabile phosphine-amide palladium and nickel catalysts for ethylene (co)polymerization with polar monomers using a cyclizing strategy. Chin. J. Polym. Sci..

[56] Cai Z., Xiao D., Do L.H. (2015). Fine-tuning nickel phenoxyimine olefin polymerization catalysts: Performance boosting by alkali cations. J. Am. Chem. Soc..

[57] Cai Z., Do L.H. (2017). Customizing polyolefin morphology by selective pairing of alkali ions with nickel phenoxyimine-polyethylene glycol catalysts. Organometallics.

[58] Tahmouresilerd B., Xiao D., Do L.H. (2021). Rigidifying cation-tunable nickel catalysts increases activity and polar monomer incorporation in ethylene and methyl acrylate copolymerization. Inorg. Chem..

[59] Chen M., Chen C. (2018). A versatile ligand platform for palladium- and nickel-catalyzed ethylene copolymerization with polar monomers. Angew. Chem. Int. Ed..

[60] Jung J., Yasuda H., Nozaki K. (2020). Copolymerization of nonpolar olefins and allyl acetate using nickel catalysts bearing a methylene-bridged bisphosphine monoxide ligand. Macromolecules.

[61] Hong C., Sui X., Li Z., Pang W., Chen M. (2018). Phosphine phosphonic amide nickel catalyzed ethylene polymerization and copolymerization with polar monomers. Dalton Trans..

[62] Zou C., Liao D., Pang W., Chen M., Tan C. (2021). Versatile PNPO ligands for palladium and nickel catalyzed ethylene polymerization and copolymerization with polar monomers. J. Catal..

[63] Brassat I., Keim W., Killat S., Möthrath M., Mastrorilli P., Nobile C.F., Suranna G.P. (2000). Synthesis and catalytic activity of allyl, methallyl and methyl complexes of nickel(II) and palladium(II) with biphosphine monoxide ligands: Oligomerization of ethylene and copolymerization of ethylene and carbon monoxide. J. Mol. Catal. A Chem..

[64] Xu M., Yu F., Li P., Xu G., Zhang S., Wang F. (2020). Enhancing chain initiation efficiency in the cationic allyl-nickel catalyzed (co)polymerization of ethylene and methyl acrylate. Inorg. Chem..

[65] Lv X., Du Y., Du S., Xiang L. (2022). Graded preparation and industrial applications of large-ball polyolefin catalyst carriers. Catalysts.

[66] Atiqullah M., Al-Asiri H.S. (2022). Polyolefin catalyst research: A product-driven industrial perspective. Chem. Rec..

[67] Culver D.B., Tafazolian H., Conley M.P. (2018). A Bulky Pd (II) α-diimine catalyst supported on sulfated zirconia for the polymerization of ethylene and copolymerization of ethylene and methyl acrylate. Organometallics.

[68] Wang Q., Sun Y., Pan Y., Pang W., Si G., Zou C. (2023). Heterogenization of nickel catalysts with ionic liquid-modified supports for ethylene polymerization and copolymerization. J. Polym. Sci..

[69] Zhang H., Zhang Z., Cai Z., Li M., Liu Z. (2022). Influence of silica-supported alkylaluminum on heterogeneous zwitterionic anilinonaphthoquinone nickel and palladium-catalyzed ethylene polymerization and copolymerization with polar monomers. ACS Catal..

[70] Zou C., Si G., Chen C. (2022). A general strategy for heterogenizing olefin polymerization catalysts and the synthesis of polyolefins and composites. Nat. Commun..

[71] Cai Z., Do L.H. (2018). Thermally robust heterobimetallic palladium-alkali catalysts for ethylene and alkyl acrylate copolymerization. Organometallics.

[72] Xiao D., Cai Z., Do L.H. (2019). Accelerating ethylene polymerization using secondary metal ions in tetrahydrofuran. Dalton Trans..

[73] Tran T.V., Karas L.J., Wu J.I., Do L.H. (2020). Elucidating secondary metal cation effects on nickel olefin polymerization catalysts. ACS Catal..

[74] Wang W., Nie N., Xu M., Zou C. (2023). Lewis acid modulation in phosphorus phenol nickel catalyzed ethylene polymerization and copolymerization. Polym. Chem..

[75] Cao L., Cai Z., Li M. (2022). Phosphinobenzenamine nickel catalyzed efficient copolymerization of methyl acrylate with ethylene and norbornene. Macromolecules.

[76] Xiong S., Shoshani M.M., Zhang X., Spinney H.A., Nett A.J., Henderson B.S., Miller T.F., Agapie T. (2021). Efficient copolymerization of acrylate and ethylene with neutral P, O-chelated nickel catalysts: Mechanistic investigations of monomer insertion and chelate formation. J. Am. Chem. Soc..

[77] Xiong S., Shoshani M.M., Nett A.J., Spinney H.A., Henderson B.S., Agapie T. (2023). Nickel-based heterometallic catalysts for ethylene-acrylate copolymerization: Interrogating effects of secondary metal additives. Organometallics.

[78] Xiong S., Hong A., Bailey B.C., Spinney H.A., Senecal T.D., Bailey H., Agapie T. (2022). Highly active and thermally robust nickel enolate catalysts for the synthesis of ethylene-acrylate copolymers. Angew. Chem. Int. Ed..

[79] Xiong S., Hong A., Ghana P., Bailey B.C., Spinney H.A., Bailey H., Henderson B.S., Marshall S., Agapie T. (2023). Acrylate-induced β-H elimination in coordination insertion copolymerizaton catalyzed by nickel. J. Am. Chem. Soc..

[80] Xiong S., Ghana P., Bailey B.C., Spinney H.A., Henderson B.S., Espinosa M.R., Agapie T. (2023). Impact of labile ligands on catalyst initiation and chain propagation in Ni-catalyzed ethylene/acrylate copolymerization. ACS Catal..

[81] Xiong S., Spinney H.A., Bailey B.C., Henderson B.S., Tekpor A.A., Espinosa M.R., Saha P., Agapie T. (2024). Switchable synthesis of ethylene/acrylate copolymers by a dinickel catalyst: Evidence for chain growth on both nickel centers and concepts of cation exchange polymerization. ACS Catal..

[82] Keim W. (2013). Oligomerization of ethylene to alpha-olefins: Discovery and development of the Shell Higher Olefin Process (SHOP). Angew. Chem. Int. Ed..

[83] Shoshani M.M., Xiong S., Lawniczak J.J., Zhang X., Miller T.F., Agapie T. (2022). Phosphine-phenoxide nickel catalysts for ethylene/acrylate copolymerization: Olefin coordination and complex isomerization studies relevant to the mechanism of catalysis. Organometallics.

[84] Zhang Y., Wang C., Mecking S., Jian Z. (2020). Ultrahigh branching of main-chain-functionalized polyethylenes by inverted insertion selectivity. Angew. Chem. Int. Ed..

[85] McInnis J.P., Delferro M., Marks T.J. (2014). Multinuclear group 4 catalysis: Olefin polymerization pathways modified by strong metal–metal cooperative effects. Accounts. Chem. Res..

[86] Yue Q., Gao R., Song Z., Lai J., Zhang R., Wang Y., Gou Q. (2024). Recent advancements in multinuclear early transition metal catalysts for olefin polymerization through cooperative effects. E-Polymers.

[87] Cai Z., Xiao D., Do L.H. (2019). Cooperative heterobimetallic catalysts in coordination insertion polymerization. Comment. Inorg. Chem..

[88] Zou C., Wang Q., Si G., Chen C. (2023). A co-anchoring strategy for the synthesis of polar bimodal polyethylene. Nat. Commun..

[89] Jin Z., Fan H., Li B.-G., Zhu S. (2016). Evaluation of octyltetramethyldisiloxane-containing ethylene copolymers as composite lubricant for high-density polyethylene. Macromol. Mater. Eng..

[90] Jin Z., Fan H., Li B.-G., Zhu S. (2016). Synthesis of a novel type of octyltetramethyldisiloxane-containing olefinic macromonomer and its copolymerization with ethylene. Polymer.

[91] Moyano M.A., París R., Martín-Martínez J.M. (2016). Changes in compatibility, tack and viscoelastic properties of ethylene n-butyl acrylate (EBA) copolymer-pentaerythritol rosin ester blend by adding microcrystalline wax, Fischer-Tropsch wax and mixture of waxes. Int. J. Adhes. Adhes..

[92] Moyano M.A., París R., Martín-Martínez J.M. (2017). Assessment of the compatibility in hot melts by using different thermoanalytical methods. Ethylene/*n*-butyl acrylate (EBA) hot melts containing tackifiers of different nature. J. Therm. Anal. Calorim..

[93] Xu M., Chen C. (2021). A disubstituted-norbornene-based comonomer strategy to address polar monomer problem. Sci. Bull..

[94] Bielawski C.W., Morita T., Grubbs R.H. (2000). Synthesis of ABA triblock copolymers via a tandem ring-opening metathesis polymerization: Atom transfer radical polymerization approach. Macromolecules.

[95] Mai B., Liu R., Li Z., Feng S., Wu Q., Gao H., Liang G., Zhu F. (2015). Synthesis and self-assembly in aqueous solution of amphiphilic diblock copolymers containing hyperbranched polyethylene. Polymer.

[96] Gao H., Hu Z., Guan Q., Liu Y., Zhu F., Wu Q. (2013). Synthesis and thermoreversible gelation of coil-helical polyethylene-block-poly(gamma-benzyl-L-glutamate) diblock copolymer. Polymer.

[97] Zhao Y., Shi X., Gao H., Zhang L., Zhu F., Wu Q. (2012). Thermo- and pH-sensitive polyethylene-based diblock and triblock copolymers: Synthesis and self-assembly in aqueous solution. J. Mater. Chem..

[98] Zhang F., Zhao Y., Wang D., Yan M., Zhang J., Zhang P., Ding T., Chen L., Chen C. (2021). Current technologies for plastic waste treatment: A review. J. Clean. Prod..

[99] Sangroniz A., Zhu J.-B., Tang X., Etxeberria A., Chen E.Y.X., Sardon H. (2019). Packaging materials with desired mechanical and barrier properties and full chemical recyclability. Nat. Commun..

[100] Roy P.K., Hakkarainen M., Varrna I.K., Albertsson A.-C. (2011). Degradable polyethylene: Fantasy or reality. Environ. Sci. Technol..

[101] De Stefano F., Baur M., De Rosa C., Mecking S. (2024). Keto-polyethylenes with controlled crystallinity and materials properties from catalytic ethylene-CO-norbornene terpolymerization. Macromolecules.

